# Abalone Collagen Extracts Potentiate Stem Cell Properties of Human Epidermal Keratinocytes

**DOI:** 10.3390/md17070424

**Published:** 2019-07-20

**Authors:** Sajee Thaweekitphathanaphakdee, Pithi Chanvorachote, Sagaw Prateepchinda, Mattaka Khongkow, Apirada Sucontphunt

**Affiliations:** 1 College of Pharmacy, Rangsit University, Pathum Thani 12000, Thailand; 2 Department of Pharmacology and Physiology, Faculty of Pharmaceutical Sciences, Chulalongkorn University, Bangkok 10330, Thailand; 3 Nation Nanotechnology Center, National Science and Technology Development Agency (NSTDA), Pathum Thani 12120, Thailand; 4 The Herbal Medicinal Products Research and Development Center, College of Pharmacy, Rangsit University, Pathum Thani 12000, Thailand

**Keywords:** abalone collagen extract, keratinocytes, stemness, wound healing, MTT

## Abstract

Stem cell activities in human tissues are critical for tissue integrity and function. Maintaining keratinocyte stem cells (KSCs) stemness helps sustain healthy skin by supporting keratinocyte renewal, involving the formation of epidermal barriers. In this study, abalone collagen (AC) extracts with molecular weights of 3 kDa (AC 1) and 300 kDa (AC 2) were compared to the epidermal growth factor (EGF) for their effects on cell proliferation, cell migration (wound healing), spheroid formation, and the expression level of stem cell markers on human keratinocytes (HaCaT cells). Cell viability was measured by 3-(4,5-dimethylthiazol-2-yl)-2,5-diphenyltetrazolium bromide (MTT) assay and cell proliferation was quantified by ATP and DNA content analysis and Sulforhodamine B (SRB) assays. Cell migration assay was determined using the scratch wound healing test. Spheroid formation was evaluated and the expression level of stem cell markers was investigated by western blot analysis. The results showed that AC 1 at the concentration of 100 µg/mL could stimulate HaCaT cell proliferation, migration, spheroid formation, and the expression level of stem cell markers (keratin 19, β-catenin, ALDH1A1) compared to the control. In conclusion, a smaller molecular weight of abalone collagen extract exhibits a better effect on keratinocytes proliferation, migration, and stemness, which could be a potential active ingredient in cosmeceutical products.

## 1. Introduction

The skin is a large human organ that renews itself throughout life in supporting the complete integrity ensuring its functions [1,2]. The skin renewal, especially epidermis, is critical for the barrier function of human skin [3,4]. In particular, the renewal process, as well as the induction of massive cell proliferation, are required in response to skin damages caused by several factors including ultraviolet, heat, and wound [5,6]. The main regulators of epidermal renewal and skin homeostasis are keratinocyte stem cells (KSCs) residing in the basal layer of epidermis and hair follicle [7]. Mature epidermis of adult skin is a stratified squamous epithelium, in which only certain cells of KSCs and their derivative progenitors at the innermost layer (stratum basale) preserve proliferative activity [8]. Such KSCs do not only produce components of extracellular matrix (ECM), but also provide cytokines and growth factors that govern the upper layer cells both for fundamental function and response to skin impairment [9]. In the aged skin, numbers and activities of KSCs were found to dramatically decrease, leading to the gradually impairment of the epidermal barrier [10].

Evidence suggests that KSCs express numerous proteins which play significant roles in retaining stem cell-like phenotypes, such as keratin 19, β-catenin, ALDH1A1, and others. Keratin 19 is a marker of skin stem cells which is found in the basal epidermal layer and the bulge region of the hair follicle. It is proportionally reduced in expression from older donors [11,12]. β-catenin plays a role in the Wnt signaling pathway and the cadherin complex which functions as a co-transcription factor of T-cell factors/lymphoid enhancing factors (TCF/LEF) [13,14]. This pathway controls stem cell renewal and regulates skin homeostasis and development [15,16]. In addition, ALDH1A1 is a detoxifying enzyme that oxidizes aldehydes and converts retinol to retinoic acid, resulting in the elimination of toxic byproducts of reactive oxygen species (ROS) and a rise in cellular antioxidant activity. A high level of ALDH1A1 activity has been proposed to be a common feature of stem cells [17,18]. An increase in stem cell-like phenotypes or stemness in keratinocytes may raise the rate of cell growth to maintain epidermal homeostasis, as well as keep the skin healthy [19,20].

Collagen is the most common protein in the skin and bone [21]. The structure of collagen is a triple helix molecule consisting of three polypeptide chains and repeating Gly-X-Y sequences. Position X and Y are often occupied by proline and 4-hydroxyproline. Polypeptide chains form a network in the extracellular matrix [22]. The collagen network helps to provide flexibility, strength to support cellular structures, and maintain skin hydration [23]. Currently, about 28 types of collagen are identified in human tissues, and approximately 80–90% of collagen is presented in the skin layer [24]. Collagen has been widely used in various applications, such as anti-aging and skin-hydrating for cosmeceuticals [25,26], scaffolds for tissue engineering [27,28,29] and drug delivery systems [30,31,32]. In the past, major sources of collagen were bovine and porcine. There are some disadvantages of these sources; for instance, forbidden usage due to religious restriction, and contamination of several transmitted diseases such as bovine spongiform encephalopathy (BSE) and transmissible spongiform encephalopathy (TSE), leading to a decrease in these use of collagen sources [26,33]. Therefore, marine collagen sources from fishes [34,35,36], sponges [37], squids [38], jellyfish [39], and others gain high interest due to no risk of transmitted diseases, their higher collagen content, low antigenicity, and a reduction in environmental waste [33].

Abalone is a valuable marine gastropod that is wildly cultured. It consists of several molecules like proteins, polysaccharides, and fatty acids. It has been used in traditional medicine and found to have multiple biological activities, such as anti-oxidant, anti-inflammatory, and anti-cancer activities [40,41]. Many studies have found that marine collagen extracts could increase cell proliferation and the migration of skin cells [35,36,42,43]. However, no research on the effects of abalone collagen on human keratinocytes has been conducted. We hypothesize that abalone collagen could increase stem cell activity of keratinocytes, as well as increase cell activity, including cell proliferation and migration. The aim of this study is to compare the effects of abalone collagen (AC) extracts with molecular weights of 3 kDa (AC 1) and 300 kDa (AC 2) to the epidermal growth factor (EGF) on cell proliferation, migration, spheroid formation, and the expression levels of stem cell markers on human keratinocytes (HaCaT cells). 

## 2. Results and Discussion

### 2.1. Effects of Abalone Collagen Extracts on Cell Viability and Cell Proliferation

**Figure 1:**
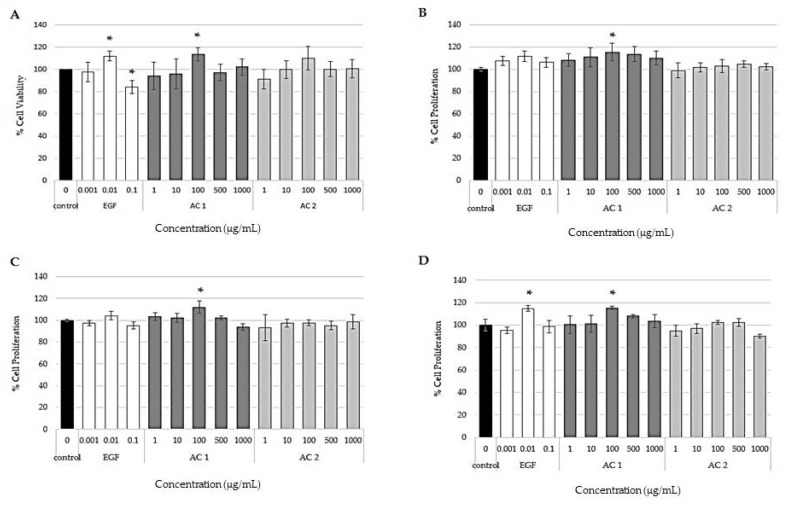
Effect of keratinocytes in response to various concentrations of abalone collagen (AC) 1, AC 2 and EGF for 48 h compared to the control by 3-(4,5-dimethylthiazol-2-yl)-2,5-diphenyltetrazolium bromide (MTT) assay (**A**), ATP assay (**B**), DNA assay (**C**) and Sulforhodamine B (SRB) assay (**D**). Data represent the means obtained from three independent experiments ± SD. * *p* < 0.05 compared to the control.

The proliferation of epidermal stem cells plays a key role in epidermis renewal and wound healing [44,45]. The effect of AC 1 and AC 2 on cell viability and cell proliferation were investigated. For cell viability assay, 3-(4,5-dimethylthiazol-2-yl)-2,5-diphenyltetrazolium bromide (MTT) assay was performed. The principle of this assay is to measure formazan crystal that is reduced by mitochondrial reductase. However, there is limitation with this assay to apply MTT assay for cell proliferation measurement [46,47]. Therefore, in this study, the number of cells was determined by quantifying ATP, DNA, and total cellular protein content to study the proliferation effect of AC 1 and AC 2. Briefly, HaCaT cells were cultivated in the presence of various concentrations of AC 1 (0–1000 µg/mL), AC 2 (0–1000 µg/mL), and EGF (1–100 ng/mL) for 48 h. In terms of stem cell biology, EGF was shown to stimulate stem cell proliferation and survival [48,49]. Besides, EGF can activate Wnt/β-catenin, a cellular signal that dominates underlying mechanism for stemness maintenance of many stem cells [50]. Therefore, EGF was used as a positive control in the present study because its direct link to the molecular mechanism of stem cell control. Figure 1A shows that AC 1 (100 µg/mL) and EGF (10 ng/mL) significantly enhanced cell viability when compared to the control, which is medium alone (*p* < 0.05). In a high concentration of EGF (100 ng/mL), the cell viability significantly decreased when compared to the control (*p* < 0.05). For cell proliferation, only AC 1 at the concentration of 100 µg/mL significantly promoted cell proliferation in ATP, DNA, and Sulforhodamine B (SRB) assays as shown in Figure 1B–D, respectively. From cell viability and cell proliferation results, 100 µg/mL of AC 1, 100 µg/mL of AC 2, and 10 ng/mL of EGF were selected for further study on their activities.

### 2.2. Abalone Collagen Extracts Induces Epithelization

**Figure 2:**
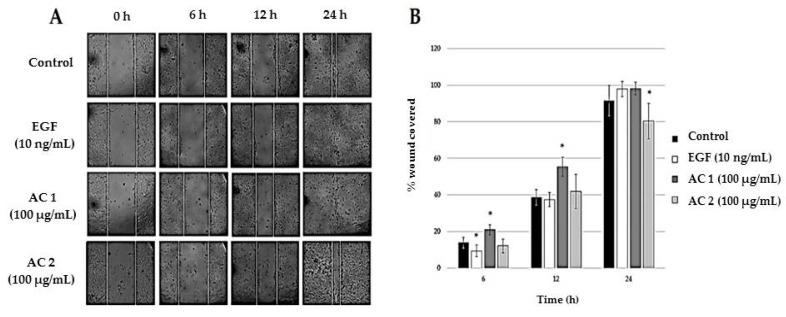
Effects of AC extracts on the scratch closure at different time points (**A**). Percentage of wound covered by cells treated with AC 1, AC 2, EGF, and the control on human keratinocytes (HaCaT cells) using a scratch test at different time points (**B**). Data represent the means obtained from three independent experiments ± SD. * *p* < 0.05 compared to the control.

Epithelization was found to link with the activity of epidermal stem cell during the wound healing process [44,51]. The cell movement activity of keratinocytes over a wounded space was futher investigated as described in Materials and Methods. The scratch test was performed in HaCaT cells treated with AC 1 (100 µg/mL), AC 2 (100 µg/mL), or EGF (10 ng/mL) in Figure 2A. The percentage of wound covered at different time points was shown in Figure 2B. At 6 and 12 h after the scratch test, AC 1 (100 µg/mL) significantly stimulated wound closure more effectively than EGF (10 ng/mL) and the control (*p* < 0.05) did. At 24 h after the scratch test, the wound covered by cells treated with AC 1 (100 µg/mL) was higher than that of the control, but similar to those treated with EGF (10 ng/mL), whereas the wound covered by those treated with AC 2 (100 µg/mL) was lower than the control (*p* < 0.05). In conclusion, AC 1 significantly stimulated cell migration (wound healing) activity faster than EGF (10 ng/mL) at 6 and 12 h after the scratch test. 

### 2.3. Abalone Collagen Extracts Potentiates 3D Spheroid Forming Activity

**Figure 3:**
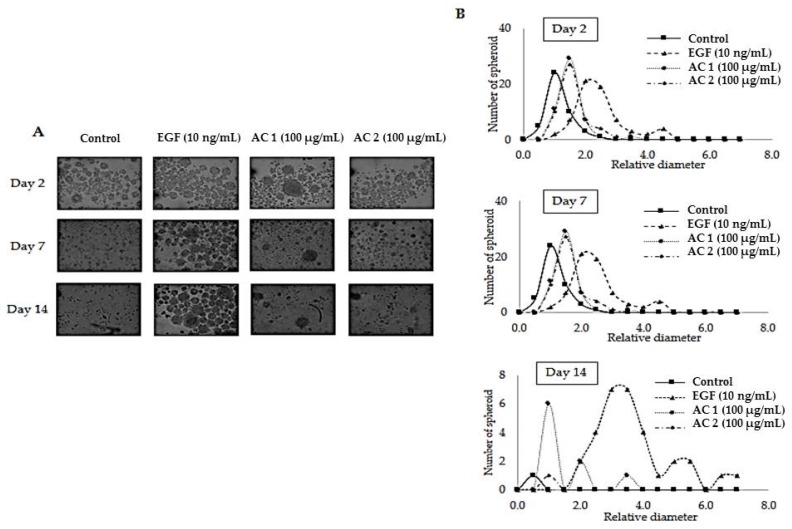
Evaluation of HaCaT cell spheroid formation induced by abalone collagen (AC) extracts: 20× phase-contrast images of spheroids on days 2, 7, and 14 (**A**), and number of spheroids and relative diameters on day 2, day 7, and day 14 of cells treated with AC 1, AC 2, EGF, and the control (**B**).

Stem cells preserve their unique property to grow in an anchorage-independent condition with superior cellular survival signals [52,53]. Therefore, the three-dimensional (3D) spheroid forming assay was utilized to evaluate the stem cell phenotypes [54,55]. Here, the ability of keratinocytes to grow and survive in 3D culture was assessed by culturing the HaCaT cells in 96-well ultra-low-attachment plates in the presence of AC 1 (100 µg/mL), AC 2 (100 µg/mL), and EGF (10 ng/mL). The cells were allowed to grow for 14 days. Phase-contrast images of spheroids are shown in Figure 3A. At day 2, cells started to form spheroids in all groups and the relative diameters of the cells treated with AC 1, AC 2, and EGF were larger than that of the control (*p* < 0.05) (Figure 3B). At day 7, the relative diameters of the cells treated with AC 1 and EGF were larger than that of the control (*p* < 0.05) (Figure 3B). In contrast, the spheroids of the control were gradually deformed. At day 14, the spheroids of the control and those treated with AC 2 were deformed and underwent apoptosis. Whereas, in EGF and AC 1 groups, spheroids still remained, but the relative diameters of EGF treated spheroids were higher than those with AC 1 treatment (*p* < 0.05) (Figure 3B). In summary, AC 1 but not AC 2 could stimulate the spheroid formation of HaCaT cells within 14 days. 

### 2.4. Evaluation of Stem Cell Markers

**Figure 4:**
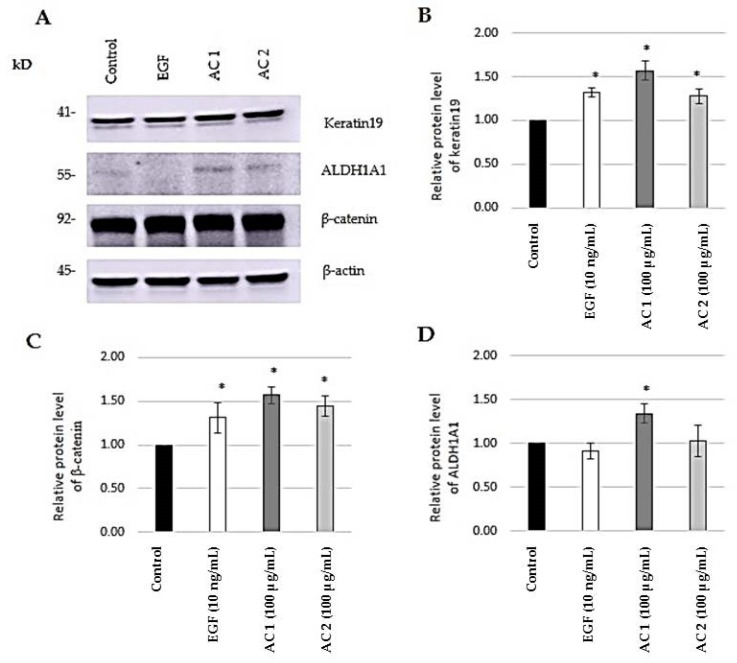
Evaluation of stem cell markers induced by abalone collagen extracts: Expression levels of stem cell markers in HaCaT cells by western blot analysis (**A**), relative protein levels of keratin 19 (**B**), relative protein levels of β-catenin (**C**) and relative protein levels of ALDH1A1 (**D**) of cells treated with AC 1, AC 2, and EGF. Data represent the means of three independent experiments ± SD. * *p* < 0.05 compared to the control.

Having found that abalone collagen extracts potentiate the stem cell-like properties in keratinocytes, we next confirmed such a finding by determining the stem cell markers in AC-treated keratinocytes. The expression levels of stem cell markers including keratin19, β-catenin, and ALDH1A1 were evaluated by western blot analysis. Keratin 19 is a marker of the skin stem cell and the smallest member of the keratin family (40 kDa). It appears in transit-amplifying (TA) cells in the basal layer and hair follicles. In addition, a cell with an increasing age proportionally reduced in keratin 19 expression [11,12,56]. β-catenin was determined as a stem cell marker in many epithelial tissues. β-catenin functions as a co-transcription factor of TCF/LE in the Wnt signaling pathway. This pathway regulates epidermal stem cell renewal and lineage selection [14,15,16,57]. ALDH1A1, considered as a marker for stem cells, plays a functional role, including cell self-protection and survival. ALDH1A1 is a member of the aldehyde dehydrogenase superfamily for cellular detoxification [17,58]. The expression levels of stem cell markers in cells treated with AC 1 (100 µg/mL), AC 2 (100 µg/mL), and EGF (10 ng/mL) were quantified by western blot analysis and ImageJ analysis (Figure 4A). All samples significantly increased the expression levels of keratin 19 and β-catenin when compared to the control (*p* < 0.05) (Figure 4B,C). The level of ALDH1A1 was significantly induced by only AC 1 when compared to the control (*p* < 0.05) (Figure 4D). No significant change in ALDH1A1 expression was found after EGF and AC 2 treatment compared to the control. Among all, AC 1 was the best at inducing the expressions of stem cell markers on HaCaT cells.

From all experiments, AC 1 (3 kDa) at a concentration of 100 µg/ml, but not AC 2 (300 kDa), increased cell proliferation, migration, spheroid formation, and the expression levels of stem cell markers of HaCaT cells when compared to the control. These results indicate an effect of abalone collagen molecular weight on keratinocyte response. Lower molecular weight molecules could penetrate into cells better than a higher molecular weight molecule, therefore leading to better cell or tissue functional improvement [59]. Furthermore, many studies have found that collagen with molecular weights of less than 5 kDa can increase cell proliferation, migration, adhesion, anti-oxidization, or angiotensin-I converting enzyme (ACE) inhibition activity [36,60,61,62]. Therefore, the molecular weight of collagen could be considered as a significant factor for collagen selection.

As the collagen was shown to potentiate stem cell properties, survival, and proliferation in some studies, we have provided the direct information for the first time that abalone collagen should induce the stem cell signal through Wnt/β-catenin signal. The study in colorectal cancer found that type I collagen could increase stem cell-like phenotype through the engagement and activation of α2β1 integrin [63]. As integrin function as a cell surface receptor that provides several survival and proliferation signals, and such signals such as AKT [64] and Wnt/β-catenin [65] activate stemness of cells, it is possible that collagen in our study may, at least in part, activate stemness through integrin- β-catenin-dependent mechanism. From the results, abalone collagen potentiates stemness properties of keratinocytes, which are important for epidermal homeostasis and skin barrier function [66,67]. Additionally, collagen from other sources, such as sponge, has been found to stimulate and increase cell growth, exhibit antioxidant activity, and protect cells from UV-induced death [37]. Abalone collagen, therefore might be useful in cosmetic formulations for photoaged skin repair, as well as improvement of skin barrier function.

## 3. Materials and Methods 

### 3.1. Materials

Abalone collagen (AC) extracts with molecular weights of 3 kDa (AC 1) and 300 kDa (AC 2) were supported by Phuket Collagen Co., Ltd. (Phuket Collagen Co., Ltd, Phuket, Thailand). The epidermal growth factor (EGF) used as a positive control was purchased from Sigma-Aldrich (Sigma-Aldrich, St. Louis, MO, USA).

### 3.2. Cell Culture

HaCaT cells obtained from the Cell Lines Service (Cell Lines Service, Eppelheim, Germany), were maintained at 37 °C under 5% CO_2_ in complete DMEM containing Dulbecco’s modified Eagle’s medium (DMEM), 10% heat-inactivated fetal bovine serum (FBS), 2 mM of L-glutamine, 100 U/mL of penicillin, and 100 U/mL of streptomycin. DMEM medium, FBS, L-glutamine, penicillin/streptomycin, phosphate-buffered saline (PBS), and trypsin-EDTA were purchased from GIBCO (Gibco, Grand Island, NY, USA).

### 3.3. Cell Viability by MTT Assay

3-(4,5-dimethylthiazol-2-yl)-2,5-diphenyltetrazolium bromide (MTT) assay was performed to analyze cell viability. HaCaT cells were seeded in 96-well plates at a density of 4000 cells/well and incubated for 24 h [68,69]. Then, cells were treated with complete DMEM containing AC 1 and AC 2 with various concentrations of 1, 10, 100, 500, and 1000 µg/mL, and EGF with concentrations of 1, 10, and 100 ng/mL, for 48 h. Then, the medium was removed and cells were added with 100 µL of MTT (Sigma-Aldrich, St. Louis, MO, USA) at the concentration of 4 mg/mL and incubated at 37 °C for 4 h. Later, the MTT solution was removed and the formazan crystals were dissolved with 100 µL of dimethylsulfoxide (DMSO). The absorbance (Abs) of MTT product was measured at 570 nm with 650 nm as a reference absorbance using a microplate reader (Benchmark^TM^ plus, Bio-Rad, Hercules, CA, USA). Percentage of cell viability was calculated by the following formula:

% cell viability = (Abs 570–650 nm of treatment/Abs 570–650 nm of control) × 100,(1)

### 3.4. Cell Proliferation by ATP content Analysis

HaCaT cells were seeded in 96-well plates at a density of 4000 cells/well and incubated for 24 h. Then, cells were treated with complete DMEM containing AC 1 and AC 2 with various concentrations of 1, 10, 100, 500, and 1000 µg/mL, and EGF with concentrations of 1, 10, and 100 ng/mL, for 48 h. Then, the medium was removed and 100 µL of CellTiter-Glo^®^ reagent (Promega, Madison, WI, USA) was added. Cells were incubated at room temperature for 10 min to stabilize luminescent signal. Luminescence was measured using microplate luminometer (SpectraMax^®^ L luminometer, Molecular Devices, Sunnyvale, CA, USA). Percentage of cell proliferation was calculated by the following formula:

% cell proliferation = (luminescence of treatment/luminescence of control) × 100,(2)

### 3.5. Cell Proliferation by DNA Assay 

HaCaT cells were seeded in 48-well plates at a density of 10,000 cells/well and incubated for 24 h. Then, cells were treated with complete DMEM containing AC 1 and AC 2 with various concentrations of 1, 10, 100, 500, and 1000 µg/mL, and EGF with concentrations of 1, 10, and 100 ng/mL, for 48 h. After washing the cells with 500 µL of 1X-PBS, the cells were incubated with 0.1% Triton X-100 (Sigma-Aldrich, St. Louis, MO, USA) for 10 min. DNA content was then analyzed using a dsDNA assay kit (Quant-iT^TM^ PicoGreen^®^, Molecular Probes, Eugene, OR, USA). Each experimental DNA solution was diluted with 30 µL of TE solution (10 mM Tris and 1 mM EDTA) to a final volume of 100 µL in 96-well plates. Then, 100 µL of the Quant-iT™ PicoGreen^®^ dsDNA reagent was added to each sample and protected from light. Later, the fluorescence intensity was measured using a microplate reader (Synergy™ H1, BioTek, Winooski, VT, USA) with fluorescence emission at 535 nm and excitation at 485 nm. The standard curve was used to determine the dsDNA concentration of each sample. Percentage of cell proliferation was calculated by the following formula:

% cell proliferation = (DNA concentration of treatment/DNA concentration of control) × 100,(3)

### 3.6. Cell Proliferation by Total Cellular Protein Content Assay (SRB Assay)

HaCaT cells were seeded in 96-well plates at a density of 4000 cells/well and incubated for 24 h. Then, cells were treated with complete DMEM containing AC 1 and AC 2 with various concentrations of 1, 10, 100, 500, and 1000 µg/mL, and EGF with concentrations of 1, 10, and 100 ng/mL, for 48 h. The cells were fixed with 100 µL of cold 40% trichloroacetic acid (TCA) solution (Sigma-Aldrich, St. Louis, MO, USA) and incubated at 4 °C for 1 h. Then, cells were rinsed with water several times and plates were air-dried. Sulforhodamine B (SRB) solution (0.04% *w*/*v*) was added to each well and cells were allowed to stain at room temperature for 1 h. Then, the cells were rinsed quickly with 1% acetic acid and allowed the plate to air-dry. Later, 100 µL of 10 mM Tris base (Biorad, Hercules, CA, USA) was added to each well. The absorbance (Abs) was measured at 510 nm using a microplate reader (Synergy™ H1, BioTek, Winooski, VT, USA). Percentage of cell proliferation was calculated by the following formula:

% cell proliferation = (Abs 510 nm of treatment/Abs 510 nm of control) × 100,(4)

### 3.7. Cell Migration (Wound Healing) by Scratch Test

HaCaT cells were seeded in 96-well plate at the density of 50,000 cells/well and allowed to grow until confluent for 24 h. A 200-µL pipette tip was used to create a straight line scratch wound on the monolayer of cells. The cells monolayer was washed twice with 100 µL of 1X-PBS and replaced with 100 µL of complete DMEM containing AC 1 (100 µg/mL), AC 2 (100 µg/mL), and EGF (10 ng/mL). The cells were incubated at 37 °C for 24 h. Evidence of cells migrating into the wound space were captured at 0, 6, 12 and 24 h after wounding by an inverted microscope (CKX41-E-330, Olympus, Tokyo, Japan). Images were analyzed using Image J software (Version 1.45, National institute of Health, Bethesda, MD, USA). Percentage of wound covered was obtained by the following formula:

% wound covered = 100 − (Width of treatment wound/Width of control wound × 100),(5)

### 3.8. Spheroid Formation 

HaCaT cells were seeded at a density of 1000 cells/well into 96-well ultra-low-attachment plates (Costar-Corning, Corning, NY, USA) with 100 µL of DMEM and 1% FBS. AC 1 (100 µg/mL), AC 2 (100 µg/mL), and EGF (10 ng/mL) was mixed in the media for this experiment. Then, 20 µL of media was added every 2–3 days. Spheroids were imaged with an inverted microscope (CKX41-E-330, Olympus, Tokyo, Japan) on days 2, 7, and 14. Images were quantitatively analyzed using Image J software and calculated for relative diameters.

### 3.9. Stem Cell Marker Quantification by Western Blot Analysis. 

HaCaT cells were seeded at a density of 2.5 × 10^5^ cells/dish onto 60 × 15 mm dishes (SPL life sciences, Gyeonggi-do, Korea) for 24 h and cultured with complete DMEM containing AC 1 (100 µg/mL), AC 2 (100 µg/mL), and EGF (10 ng/mL) for 48 h. After washing the cells twice in 1X-PBS, the cells were incubated with ice-cold lysis buffer containing Ripa lysis buffer (Amresco^®^, Solon, OH, USA), 0.5% Triton X-100 (Sigma-Aldrich, St. Louis, MO, USA), water, and protease inhibitor cocktail (Amresco^®^, Solon, OH, USA) for 20 min on ice. Protein content was then analyzed using bicinchoninic acid (BCA) and a protein assay kit (Sigma-Aldrich, St. Louis, MO, USA). Equal amounts of proteins (1500 μg) were heated at 95 °C for 5 min with blue loading buffer (New England Biolabs, Beverly, MA, USA). The protein samples were loaded on 10% SDS-PAGE gels (Mini-PROTEAN^®^ TGX™, Bio-Rad, Hercules, CA, USA) before transferred to 0.2 um PVDF membranes (Bio-Rad, Hercules, CA, USA). Next, the transferred membranes were blocked in TBST buffer (25 mM Tris-HCl (pH 7.5), 125 mM NaCl, and 0.1% Tween-20) containing 5% nonfat dry milk powder (Bio-Rad, Hercules, CA, USA) for 1 h and incubated overnight with specific primary antibodies against keratin 19, β-catenin, ALDH1A1, and β-actin (Cell Signaling Technology, Boston, MA, USA). Later, membranes were washed three times with TBST and incubated with horseradish peroxidase labeled secondary antibodies goat anti-rabbit IgG (Invitrogen Life Technologies, Carlsbad, CA, USA) for 1–2 h at a room temperature. The membranes were washed three times in TBST and the immune complexes were analyzed by using HPR chemiluminescent substrate reagent kit (Invitrogen, Carlsbad, CA, USA) and detected by digital imaging with a charge-coupled device (CCD) camera-based imager (Amersham^TM^ Imager 600, GE Healthcare, Buckinghamshire, UK). A relative band density was measured using Image J program, calculated for a normalization factor as a ratio of β-actin in the corresponding samples. The densities of the samples were quantitatively compared using western blot analysis.

### 3.10. Statistical Analysis

All experiments were performed in three independent experiments. Each experiment was carried out in triplicate. Results from images were quantified by Image J analysis. Results were expressed as mean ± standard deviation (SD), which were subjected to statistical comparison using one-way ANOVA with post-hoc test at a significance level (α) of 0.05 (IBM SPSS version 21, IBM corp, Armonk, NY, USA ).

## 4. Conclusions

A smaller size of abalone collagen extract with a molecular weight of 3 kDa could significantly stimulate cell proliferation, migration, spheroid formation, and the expression levels of stem cell markers, which are key factors for functional keratinocyte stem cells. Abalone collagen extract, therefore, is a potential alternative source of collagen for cosmeceutical industry.
